# Community Pharmacists’ Acceptance of Telemedicine-Enabled Medication Dispensing in Jordan: A Mixed-Methods Study of Patient Safety Concerns, Implementation Barriers, and Required Safeguards

**DOI:** 10.3390/healthcare14101346

**Published:** 2026-05-14

**Authors:** Hayam A. AlRasheed, Wael Abu Dayyih, Zekrayat J. H. Merdas, Walid L. Wadi, Abdelrahman Alharazneh, Raed Shudifat, Anas Abed

**Affiliations:** 1 Department of Pharmacy Practice, College of Pharmacy, Princess Nourah bint Abdulrahman University, P.O. Box 84428, Riyadh 11671, Saudi Arabia; haalrasheed@pnu.edu.sa; 2 Faculty of Pharmacy, Mutah University, Al Karak 61710, Jordan; wabudayyih@mutah.edu.jo; 3 Pharmacy Department, College of Pharmacy, Amman Arab University, Amman 11953, Jordan; th.merdas@aau.edu.jo; 4 Department of Internal Medicine, College of Medicine, Mutah University, Al Karak 61710, Jordan; waleed.wadi@mutah.edu.jo; 5 College of Medicine, Mutah University, Al Karak 61710, Jordan; haraznehm@mutah.edu.jo; 6 Faculty of Nursing, Mutah University, Al Karak 61710, Jordan; rshdefat@mutah.edu.jo; 7 Department of Biopharmaceutics and Clinical Pharmacy, Faculty of Pharmacy, Al-Ahliyya Amman University, Amman 19328, Jordan

**Keywords:** telepharmacy, telemedicine, medication dispensing, patient safety, pharmacists’ acceptance, Jordan, mixed-methods

## Abstract

**Highlights:**

**What are the main findings?**

- Jordanian community pharmacists showed low acceptance of the current telemedicine-enabled medication dispensing and delivery model, with resistance driven mainly by patient safety concerns, unclear legal liability, and limited system readiness.
- Acceptance was conditional rather than absolute; pharmacists strongly supported safeguards such as mandatory pharmacist verification, direct pharmacist–patient communication, clear legal protections, and standardized delivery and dispensing protocols.

**What are the implications of the main findings?**

- Regulatory approval alone is insufficient for successful implementation; telemedicine-enabled dispensing must be supported by pharmacist oversight, legal clarity, infrastructure readiness, and robust patient safety mechanisms.
- Co-designed regulatory refinement and phased pilot implementation may improve professional acceptance and help translate telepharmacy policy into safe, acceptable, and equitable practice in Jordan.

**Abstract:**

**Background/Objectives**: Telemedicine-enabled medication dispensing and delivery was formally regulated in Jordan in 2025, but the Jordan Pharmacists Association publicly rejected the pharmacy-related provisions because of concerns about safety, liability, and the pharmacist’s professional role. This study evaluated community pharmacists’ acceptance of the regulated model and identified perceived patient safety risks, implementation barriers, and required safeguards. **Methods**: A convergent mixed-methods design was used. A cross-sectional online survey was completed by 350 licensed community pharmacists (response rate 83.3%). The questionnaire assessed willingness to participate, perceived patient safety risks, implementation barriers, and facilitators using 5-point Likert scales. Multivariable logistic regression examined predictors of willingness. Semi-structured interviews were conducted with 22 purposively sampled pharmacists until thematic saturation. Quantitative and qualitative findings were integrated using joint displays. **Results**: Only 28.3% of pharmacists were willing to participate under current conditions, 46.9% were unwilling, and 24.9% expressed conditional acceptance; 52.0% opposed national implementation. Patient safety concerns were great (mean 4.4 ± 0.6/5), especially regarding remote patient assessment (91.4%) and medication errors (88.9%). Implementation barriers were severe (mean 4.5 ± 0.5/5), mainly regulatory ambiguity (92.0%) and unclear liability (89.7%). Facilitators were strongly endorsed (mean 4.7 ± 0.4/5), particularly mandatory pharmacist verification (94.6%) and clear legal protections (93.4%). Qualitative findings reinforced pharmacists’ role as the “final safety checkpoint” and showed acceptance depended on strong safeguards. **Conclusions**: Jordanian pharmacists showed principled resistance to the current model. Acceptance depends on pharmacist oversight, legal clarity, and infrastructure readiness.

## 1. Introduction

The rapid integration of digital health technologies has transformed healthcare delivery worldwide, enabling novel models of care such as telemedicine and telepharmacy, electronic prescribing, remote counseling, and medication delivery services [1,2,3,4]. Telemedicine facilitates remote clinical consultations, diagnosis, and monitoring, while telepharmacy extends pharmaceutical care through electronic prescription processing, medication review, patient counseling, and professional support without requiring direct physical presence [2,3]. These models may improve access to healthcare and pharmaceutical services, particularly for patients in rural areas, those with mobility limitations, and individuals with chronic diseases requiring ongoing medication management [4,5].

However, it is important to distinguish general telepharmacy from the specific model examined in the present study. In its broadest sense, telepharmacy may include remote counseling, medication review, adherence support, and pharmacist-led follow-up. By contrast, telemedicine-enabled medication dispensing and delivery refers here to a regulated, platform-mediated workflow in which a remote medical consultation is linked to prescription transmission, pharmacist verification, dispensing, and delivery of medicines to the patient. This model is therefore not simply a digital extension of counseling; it changes the medication-use pathway by introducing remote clinical assessment, electronic prescription transfer, platform coordination, and medication transport. These changes raise distinct concerns related to patient safety, professional accountability, pharmacist–patient communication, medication storage, delivery traceability, and the preservation of the pharmacist’s clinical role [6,7,8,9].

Globally, telepharmacy has been associated with improved access to pharmaceutical services, enhanced medication adherence, and increased healthcare efficiency [5]. However, implementation of telepharmacy, particularly when integrated with medication dispensing and delivery systems, introduces important challenges related to patient safety, regulatory oversight, and professional accountability [6,7]. Unlike traditional pharmacy practice, remote dispensing workflows may reduce opportunities for direct pharmacist–patient interaction, potentially affecting medication verification, identification of drug-related problems, and the quality of patient counseling [2,8,9]. In addition, medication delivery systems create logistical concerns, including appropriate storage conditions, cold-chain maintenance, traceability, and secure transfer of medicines to the intended recipient [9].

The implementation of digital health innovations is shaped not only by technological availability, but also by organizational readiness, implementation climate, professional acceptance, workflow compatibility, regulatory preparedness, and perceived value to intended users. Implementation frameworks such as the Non-adoption, Abandonment, Scale-up, Spread, and Sustainability framework, the Consolidated Framework for Implementation Research, organizational readiness theory, and innovation implementation theory emphasize that health technologies may fail to become normalized in practice when they are misaligned with users’ values, available resources, clinical workflows, institutional support, or external regulatory conditions [10,11,12]. These perspectives are particularly relevant to telemedicine-enabled dispensing, where adoption depends on the interaction between digital platforms, pharmacy infrastructure, legal responsibility, professional norms, and patient safety safeguards.

Another important but underexplored dimension is pharmacists’ perception of automation within platform-mediated dispensing workflows. Telemedicine-enabled dispensing does not necessarily imply fully automated dispensing; however, it may involve automated prescription routing, electronic verification prompts, platform-generated documentation, delivery assignment, and digital tracking. Such functions may support standardization and traceability, but pharmacists may also perceive automation as a source of risk if it reduces professional discretion, encourages over-reliance on system outputs, weakens direct clinical verification, or diffuses accountability when errors occur [13]. Therefore, automation is relevant to the conceptual framing of platform-mediated dispensing, particularly because medication dispensing remains a safety-critical process requiring professional judgment.

In Jordan, digital health initiatives have expanded rapidly in recent years, including telemedicine and medication-delivery services [14,15,16]. National strategic efforts culminated in the issuance of formal instructions in 2025 regulating remote dispensing and transport of medicines. These instructions define conditions for platform-based dispensing, prohibit remote dispensing of high-risk and controlled medicines, require pharmacist involvement, and impose documentation, tracking, and delivery requirements. While representing an important regulatory step, the provisions have sparked substantial professional controversy. The Jordan Pharmacists Association publicly rejected the pharmacy-related components, citing insufficient alignment with the profession, the absence of a unified national medical record system, and concerns that the model reduces pharmacists to a transactional role rather than therapeutic partners. This controversy indicates that the debate in Jordan is no longer simply about acceptance of telepharmacy as a concept, but about the practical, legal, and professional implications of implementing a specific regulated model of remote dispensing.

Previous studies in Jordan have reported moderate awareness and acceptance of telepharmacy as a general concept, alongside concerns about medication safety, communication, and regulation [17,18]. However, these studies used cross-sectional quantitative designs and examined telepharmacy mainly as a broad service concept rather than pharmacists’ responses to a newly regulated, platform-mediated dispensing and delivery model in a live policy context.

Therefore, an important gap remains in understanding how community pharmacists perceive this specific model, particularly in relation to patient safety, legal responsibility, workflow integration, infrastructure readiness, and required safeguards. This gap is critical because pharmacists serve as the final checkpoint in the medication-use process. Given the current regulatory framework and ongoing professional resistance, a convergent mixed-methods approach is needed because quantitative data can estimate acceptance levels and identify associated factors, while qualitative data can explain the professional reasoning, safety concerns, and implementation conditions underlying those patterns.

Accordingly, this study aimed to evaluate community pharmacists’ acceptance of telemedicine-enabled medication dispensing and delivery in Jordan, identify factors associated with acceptance, and explore perceived patient safety risks, implementation barriers, and required safeguards using a convergent mixed-methods design.

## 2. Materials and Methods

### 2.1. Study Design

This study employed a convergent mixed-methods design to assess community pharmacists’ acceptance of telemedicine-enabled medication dispensing and delivery in Jordan and to identify perceived patient safety risks, implementation barriers, and required safeguards. Quantitative and qualitative data were collected concurrently, analysed separately, and integrated at the interpretation stage. Reporting followed the Strengthening the Reporting of Observational Studies in Epidemiology (STROBE) guideline for the quantitative component, the Consolidated Criteria for Reporting Qualitative Research (COREQ) for the qualitative component, and the Good Reporting of A Mixed Methods Study (GRAMMS) framework for the overall design (Supplementary Materials S1).

### 2.2. Study Setting and Policy Context

The study was conducted in the community pharmacy sector in Jordan during the period immediately following the issuance of national instructions in 2025 regulating telemedicine-enabled medication dispensing and delivery. These instructions permitted remote dispensing through digital platforms under defined conditions, required pharmacist involvement, prohibited remote dispensing of high-risk and controlled medicines, and mandated documentation, traceability, and delivery standards. Data collection occurred between December 2025 and February 2026, providing a real-time policy-contextualised evaluation of pharmacists’ responses to an active regulatory framework.

### 2.3. Quantitative Component

#### 2.3.1. Participants and Recruitment

The quantitative component targeted licensed community pharmacists actively involved in medication dispensing and patient counselling. Pharmacists not currently practising in community settings were excluded. Participants were recruited via direct outreach to pharmacies and dissemination through professional networks. The survey was hosted on a secure online platform. Respondents reviewed an electronic participant information sheet and provided informed consent. Duplicate submissions were prevented by platform settings and manual screening for identical demographic and response patterns.

#### 2.3.2. Sample Size

The target sample size for the quantitative component was set at 350 community pharmacists a priori based on both precision-based and regression-related considerations.

For descriptive estimation, assuming the most conservative expected proportion of 50%, a 95% confidence level, and a desired margin of error of approximately 5.3%, the required sample size was estimated using the single-proportion formula:

n = Z^2^p(1 − p)/d^2^. (1)

This yielded a minimum sample of approximately 342 participants. Therefore, the achieved sample of 350 was considered sufficient to provide reasonably precise descriptive estimates. For the planned multivariable logistic regression, willingness to participate was dichotomized as willing versus not willing/conditional. With 99 pharmacists classified as willing to participate and seven prespecified predictors entered into the final model, the analysis provided approximately 14 outcome events per predictor variable, exceeding the commonly used minimum threshold of 10 events per predictor. The sample was also considered adequate to support subgroup comparisons and assessment of the internal consistency of the multi-item study scales.

#### 2.3.3. Instrument Development

The survey instrument was developed through a multi-stage process informed by the literature on telepharmacy, digital health implementation, medication safety, and healthcare technology adoption [10,19,20,21,22]. Items were generated to address willingness to participate, perceived patient safety risks, implementation barriers, and required safeguards. The initial pool was reviewed by the research team and then by five experts in clinical pharmacy, medication safety, digital health, and implementation science for relevance, clarity, and contextual appropriateness. Face validity was assessed via cognitive debriefing with a pilot group of community pharmacists (n = 12). The questionnaire was revised accordingly; pilot responses were excluded from analysis.

The final survey (Supplementary Materials S2) was administered in English, the language of pharmacy education in Jordan. To accommodate varying English proficiency, participants could request Arabic clarification of any item during completion.

#### 2.3.4. Measures and Scoring

The primary outcome was willingness to participate in telemedicine-enabled medication dispensing and delivery (yes, no, or conditional acceptance). For the main logistic regression model, this was dichotomised as willing versus not willing/conditional. Support for national implementation was measured on a three-point ordinal scale (support, neutral/uncertain, oppose).

Perceived patient safety risks (8 items), implementation barriers (9 items), and facilitators/safeguards (7 items) were assessed using five-point Likert scales (1 = strongly disagree to 5 = strongly agree). Composite scores were calculated as the mean of completed items within each construct (requiring ≥80% completion). Demographic and professional characteristics were also collected.

Awareness-related variables included general familiarity with telemedicine, awareness of recent regulatory or policy discussions in Jordan regarding telemedicine-enabled dispensing, awareness of the concept of telemedicine-enabled medication dispensing and delivery, self-rated operational understanding, and awareness of specific regulatory or policy initiatives in Jordan related to this model. The policy-related awareness items were intentionally separated to distinguish general awareness of ongoing public or professional discussion from awareness of specific formal regulatory initiatives.

#### 2.3.5. Validity and Reliability

Content validity was established through expert panel review. Face validity was confirmed via pilot cognitive debriefing. Internal consistency was evaluated using Cronbach’s alpha. The patient safety concern scale demonstrated excellent reliability (α = 0.89), the implementation barrier scale demonstrated excellent reliability (α = 0.91), and the facilitator/safeguard scale demonstrated very good internal consistency (α = 0.88). All scales exceeded the 0.70 threshold. Composite reliability values were consistent with Cronbach’s alpha.

#### 2.3.6. Data Management and Statistical Analysis

Data were analysed using IBM SPSS Statistics Version 28.0. Descriptive statistics summarised participant characteristics and responses. Bivariate associations were examined using chi-square tests, Fisher’s exact tests, *t*-tests, or Mann–Whitney U tests as appropriate. Multivariable logistic regression identified independent predictors of willingness to participate; candidate variables were selected a priori on theoretical and empirical grounds. The dependent variable was dichotomised as willing versus not willing/conditional. The number of variables entered into the final model was kept limited to maintain model stability in relation to the number of outcome events. Multicollinearity among predictors was assessed using variance inflation factors (VIFs) and tolerance values before model interpretation. No problematic multicollinearity was observed, as all VIF values were below the conventional threshold of 5 and tolerance values exceeded 0.20. To examine the robustness of the findings, alternative model specifications were explored, including models excluding either age or years of professional experience because of their conceptual overlap, and models including age × experience and pharmacy type × practice location interaction terms. These sensitivity analyses did not materially alter the direction or interpretation of the main findings. Stepwise regression procedures were not used for the primary analysis because the final model was theory-driven and based on prespecified predictors; however, backward elimination was explored as a sensitivity analysis and produced a comparable pattern of significant predictors. Model fit was assessed with the Hosmer–Lemeshow test and discrimination with the area under the receiver operating characteristic curve. Statistical significance was set at *p* < 0.05 (two-sided).

### 2.4. Qualitative Component

#### 2.4.1. Design and Sampling

A descriptive qualitative approach was used to explain quantitative patterns. Participants were purposively sampled to ensure maximum variation in years of professional experience, pharmacy ownership type (independent versus chain), practice location (urban versus rural/suburban), and self-reported level of acceptance from the quantitative survey. Potential interview participants were approached by phone, email, or direct message through professional networks, and some were invited through follow-up contact after completing the quantitative survey and indicating willingness to be interviewed. Of 30 pharmacists invited, 22 completed interviews and 8 declined (mainly due to time constraints). The 22 interviewees represented variation in sex, years of experience, pharmacy type, practice location, and level of acceptance of telemedicine-enabled dispensing. Recruitment continued until thematic saturation was reached, defined as the point at which additional interviews yielded no substantively new themes or insights.

#### 2.4.2. Interview Guide Development

The semi-structured interview guide (Supplementary Materials S3) was developed iteratively in parallel with the survey instrument. It was informed by the same conceptual domains (patient safety, legal and professional responsibility, infrastructure readiness, workflow implications, and conditions for acceptance). The guide was reviewed by qualitative methods experts and piloted with three community pharmacists to assess clarity, flow, sensitivity, and feasibility. Revisions focused on improving question sequencing and encouraging concrete examples. No repeat interviews were conducted.

#### 2.4.3. Data Collection and Transcription

Interviews were conducted individually in private settings (either online through secure platforms or in person) according to participant preference. A male researcher with a background in pharmacy practice and health services research, who was an academic faculty member with a PhD degree with prior training and experience in qualitative interviewing, conducted all interviews. No prior personal or professional relationship existed between the interviewer and participants. Before each interview, participants were informed that the interviewer was an academic researcher in pharmacy practice, that the study was being conducted for research purposes only, and that he was not affiliated with regulatory authorities or any enforcement body. No non-participants were present.

Interviews lasted approximately 30–45 min and were conducted in Arabic. With written or verbal consent, all interviews were audio-recorded. Field notes were taken during and immediately after each interview to capture contextual observations, non-verbal or interactional cues where relevant, and early analytic reflections. Audio recordings were transcribed verbatim by the primary researcher. Transcripts were checked against the recordings for accuracy and de-identified before analysis. Full transcripts were not returned to participants because the study used summary-based participant checking rather than transcript validation; however, selected participants were invited to review a concise summary of the emerging themes to assess interpretive accuracy.

#### 2.4.4. Qualitative Analysis

Reflexive thematic analysis was conducted following Braun and Clarke’s six-phase approach using a hybrid inductive–deductive framework. Deductive sensitising concepts were drawn from implementation science domains, while inductive coding allowed Jordan-specific issues to emerge. Two researchers independently coded an initial subset of transcripts to develop and refine the coding framework. After stabilisation of the codebook, the remaining transcripts were coded by the primary researcher, with regular analytic meetings to discuss discrepancies and strengthen theme coherence. Coding and data management were performed in NVivo Release 14 (QSR International, Melbourne, Australia). Themes were reviewed iteratively for internal homogeneity and external heterogeneity before finalisation. A structured coding framework was developed iteratively, consisting of higher-order themes and related subthemes; a summary coding tree is provided in Supplementary Materials S4.

#### 2.4.5. Trustworthiness and Reflexivity

Credibility was enhanced through triangulation with quantitative findings, analyst debriefing, and participant-checking of theme summaries with selected participants. analyst debriefing, and summary-based participant checking. After preliminary themes were developed, a concise summary of the emerging themes and interpretations was shared with a purposive subset of interview participants representing different levels of acceptance and practice settings. Participants were asked whether the themes accurately reflected their experiences and whether any important perspectives were missing or misrepresented. Feedback was used to assess interpretive resonance and to refine theme wording, but not to quantify agreement or replace the analytic judgment of the research team. No major contradictory feedback was received, and participant comments supported the credibility and contextual relevance of the final themes.

Dependability was supported by a detailed audit trail documenting all sampling, data collection, coding, and analytic decisions. Confirmability was strengthened by reflexive memoing throughout the process and explicit discussion of alternative interpretations. Transferability was facilitated by rich contextual descriptions of participants and settings.

The research team’s professional background in pharmacy practice and health services research provided contextual insight but also introduced the potential for privileging medication safety and professional autonomy concerns. The researchers therefore acknowledged that their prior professional assumptions could shape data interpretation. This was actively mitigated through ongoing reflexive memoing, team discussions that challenged emerging assumptions, and the deliberate design of the interview guide to elicit both supportive and critical perspectives.

### 2.5. Integration of Quantitative and Qualitative Findings

Integration occurred after separate analyses using joint displays to compare quantitative distributions with qualitative themes. Meta-inferences identified convergence, complementarity, and divergence, with interpretation grounded in the 2025 regulatory framework.

### 2.6. Ethical Considerations

Ethical approval was obtained from the Institutional Review Board at Al-Ahliyya Amman University (IRB No. AAU/3/3/2025-2026). The study adhered to the Declaration of Helsinki. Participation was voluntary; informed consent was obtained electronically for the survey and verbally or in writing for interviews. All data were stored securely and accessed only by the research team.

## 3. Results

### 3.1. Characteristics of Participating Pharmacists

The survey was distributed to 420 licensed community pharmacists through direct outreach to pharmacies and professional networks. A total of 350 community pharmacists provided complete responses, yielding a response rate of 83.3%. The demographic and professional characteristics of the participants are summarised in Table 1.

The mean age of participants was 32.8 years (SD = 7.4), with the majority aged between 26 and 35 years (56.3%). Females represented 58.9% (n = 206) of the sample, while males accounted for 41.1% (n = 144), consistent with the current gender distribution of community pharmacists in Jordan.

Regarding professional experience, most participants had less than 10 years of experience (61.4%, n = 215), while 27.7% (n = 97) had between 10 and 20 years of experience, and 10.9% (n = 38) had more than 20 years of experience. The majority were employed in independent community pharmacies (72.6%, n = 254), whereas 27.4% (n = 96) worked in chain pharmacy settings.

Most pharmacists practiced in urban areas (79.1%, n = 277), reflecting the concentration of pharmacies in major cities such as Amman, Irbid, and Zarqa. The remaining 20.9% (n = 73) practiced in suburban or rural areas.

In terms of workload, 44.0% (n = 154) reported dispensing between 50 and 100 prescriptions per day, while 32.3% (n = 113) dispensed fewer than 50 prescriptions daily, and 23.7% (n = 83) dispensed more than 100 prescriptions per day.

Regarding the general familiarity with digital health concepts, 63.4% (n = 222) reported being familiar with the concept of telemedicine, while 36.6% (n = 128) reported limited or no familiarity. Additionally, 41.7% (n = 146) reported prior experience handling prescriptions received electronically (e.g., scanned prescriptions or prescriptions transmitted via electronic communication), although a formal national telemedicine-linked dispensing system had not yet been implemented. Furthermore, 34.9% (n = 122) reported awareness of recent regulatory or policy discussions in Jordan related to telemedicine-enabled medication dispensing.

### 3.2. Awareness and Knowledge of Telemedicine-Enabled Medication Dispensing and Delivery

Overall, 61.7% (n = 216) of participating pharmacists reported being aware of the concept of telemedicine-enabled medication dispensing and delivery (Table 2). When asked about their level of understanding, only 18.6% (n = 65) reported having a good understanding of how such systems operate, whereas 43.1% (n = 151) reported having a basic understanding and 38.3% (n = 134) reported having little or no understanding of the operational processes involved. Awareness of specific regulatory or policy initiatives in Jordan was lower than general awareness of recent policy discussions, with only 29.4% (n = 103) reporting awareness of formal initiatives related to telemedicine-enabled medication dispensing and delivery.

Regarding sources of information, the most commonly reported sources were professional colleagues (46.9%, n = 164), social media platforms and online discussions (42.0%, n = 147), and professional organizations or regulatory announcements (28.6%, n = 100). Fewer pharmacists reported obtaining information through formal training programs (12.9%, n = 45) or continuing professional education activities (15.7%, n = 55).

Importantly, only 29.4% (n = 103) of pharmacists reported being aware of specific regulatory or policy initiatives in Jordan related to telemedicine-enabled medication dispensing and delivery, while the majority (70.6%, n = 247) were either unaware or uncertain about current or planned regulatory frameworks.

Pharmacists practicing in urban areas demonstrated significantly higher awareness compared to those practicing in rural or suburban areas (66.8% vs. 41.1%, *p* < 0.001). Similarly, pharmacists working in chain pharmacies reported higher awareness levels compared to those in independent pharmacies (71.9% vs. 57.5%, *p* = 0.012).

Overall, these findings indicate moderate awareness of telemedicine-enabled medication dispensing and delivery among community pharmacists in Jordan, but limited depth of operational understanding and regulatory familiarity.

### 3.3. Acceptance and Willingness to Adopt Telemedicine-Enabled Medication Dispensing and Delivery

Overall, a majority of participating pharmacists expressed reservations regarding telemedicine-enabled medication dispensing and delivery. Two separate acceptance-related outcomes were assessed: pharmacists’ personal willingness to participate under current conditions and their broader position regarding national implementation of the model in its current form (Table 3). Only 28.3% (n = 99) indicated that they would be willing to participate in such systems under current conditions, whereas 46.9% (n = 164) reported that they would not be willing to participate. The remaining 24.9% (n = 87) indicated conditional acceptance, stating that their willingness would depend on the presence of clear regulatory safeguards, defined professional responsibilities, and appropriate patient safety measures.

When asked about their level of support for implementing telemedicine-enabled dispensing at a national level, 52.0% (n = 182) reported opposing the implementation in its current form, while 31.7% (n = 111) expressed neutral or uncertain positions, and only 16.3% (n = 57) reported clear support.

Pharmacists practicing in independent pharmacies were significantly more likely to oppose implementation compared to those in chain pharmacies (55.9% vs. 41.7%, *p* = 0.018). Similarly, pharmacists with more than 10 years of professional experience demonstrated higher opposition rates compared to those with fewer years of experience (58.4% vs. 43.2%, *p* = 0.011).

Urban pharmacists demonstrated slightly higher acceptance rates compared to rural pharmacists (30.7% vs. 19.2%), although opposition remained the dominant response across both groups.

Among pharmacists expressing opposition, the most frequently cited concerns included potential patient safety risks (72.6%), unclear legal and professional liability (68.3%), reduced direct pharmacist–patient interaction (61.0%), and concerns regarding inappropriate medication dispensing without adequate clinical evaluation (57.9%).

These findings indicate substantial resistance among community pharmacists toward telemedicine-enabled medication dispensing and delivery in Jordan, with acceptance primarily conditional upon the implementation of robust regulatory frameworks and patient safety safeguards.

### 3.4. Perceived Patient Safety Risks Associated with Telemedicine-Enabled Medication Dispensing and Delivery

Overall, pharmacists expressed very high levels of concern regarding potential patient safety risks associated with telemedicine-enabled medication dispensing and delivery systems (Table 4). The vast majority of participants (88.9%, n = 311) agreed or strongly agreed that such systems could increase the risk of medication errors if implemented without appropriate safeguards.

The most frequently reported safety concern was the inability to adequately assess patients and verify the appropriateness of prescribed medications without direct interaction, reported by 91.4% (n = 320) of pharmacists. Additionally, 89.1% (n = 312) expressed concern that remote dispensing may limit pharmacists’ ability to identify potential drug–drug interactions, contraindications, or inappropriate dosing.

Concerns related to the quality and effectiveness of pharmacist–patient communication were also prominent, with 86.6% (n = 303) indicating that reduced direct interaction could negatively impact patient counseling and medication safety. Similarly, 84.9% (n = 297) expressed concerns regarding unclear accountability and legal responsibility in the event of medication errors occurring within telemedicine-enabled dispensing workflows.

Logistical concerns related to medication storage and transportation were also highly prevalent. A total of 82.3% (n = 288) of pharmacists reported concerns regarding improper storage conditions during delivery, particularly for temperature-sensitive medications such as insulin and biologic therapies. Additionally, 79.4% (n = 278) expressed concern regarding the potential for dispensing errors due to incomplete or unclear electronic prescription information.

Pharmacists with more than 10 years of experience reported significantly higher safety concern scores compared to those with fewer years of experience (mean score 4.6 vs. 4.2 on a 5-point Likert scale, *p* = 0.004). Similarly, pharmacists practicing in independent pharmacies reported slightly higher concern levels compared to those in chain pharmacies.

Overall, these findings demonstrate widespread and substantial patient safety concerns among community pharmacists regarding telemedicine-enabled medication dispensing and delivery, highlighting the need for robust regulatory frameworks, clear accountability structures, and standardized safety protocols prior to implementation.

### 3.5. Perceived Implementation Barriers to Telemedicine-Enabled Medication Dispensing and Delivery

Pharmacists reported very severe implementation barriers that could hinder the safe and effective adoption of telemedicine-enabled medication dispensing and delivery systems. The most frequently reported barrier, as elucidated in Table 5, was the lack of clear regulatory frameworks defining pharmacists’ roles, responsibilities, and legal liability, identified by 92.0% (n = 322) of participants.

Similarly, 89.7% (n = 314) of pharmacists reported concerns regarding unclear professional accountability in the event of medication errors occurring during remote dispensing and delivery processes. A substantial proportion of participants (87.4%, n = 306) also reported concerns regarding the absence of standardized protocols governing prescription verification, documentation, and pharmacist counseling in telemedicine-linked dispensing workflows.

Infrastructure-related barriers were also highly prevalent. A total of 84.6% (n = 296) of pharmacists reported insufficient technological infrastructure to support safe and reliable telemedicine dispensing systems, including lack of integrated electronic prescription platforms and secure communication systems. Additionally, 82.0% (n = 287) reported concerns regarding the absence of standardized delivery protocols ensuring proper medication handling, storage conditions, and traceability during transportation.

Operational and workflow-related concerns were also prominent. Approximately 79.7% (n = 279) of pharmacists indicated that telemedicine-enabled dispensing could disrupt established pharmacy workflows, while 76.9% (n = 269) reported concerns regarding increased workload and administrative burden associated with managing electronic prescriptions and remote dispensing processes.

Economic concerns were also reported, with 73.4% (n = 257) expressing concerns that telemedicine-enabled dispensing and delivery models could negatively impact community pharmacy sustainability, particularly among independently owned pharmacies.

Pharmacists practicing in independent pharmacies reported significantly higher concern levels regarding economic and operational barriers compared to those working in chain pharmacy settings (*p* = 0.009). Similarly, pharmacists practicing in rural or suburban areas reported greater concerns regarding infrastructure limitations compared to those practicing in urban areas (*p* = 0.015).

Overall, pharmacists perceived substantial structural, regulatory, operational, and economic barriers to the implementation of telemedicine-enabled medication dispensing and delivery, emphasizing the need for comprehensive regulatory frameworks, infrastructure development, and clearly defined professional responsibilities prior to implementation.

### 3.6. Factors Associated with Acceptance of Telemedicine-Enabled Medication Dispensing and Delivery

Multivariable logistic regression analysis was conducted to identify factors independently associated with pharmacists’ willingness to participate in telemedicine-enabled medication dispensing and delivery systems. The dependent variable was defined as willingness to participate (yes vs. no/conditional).

The analysis (shown in Table 6) demonstrated that younger pharmacists were significantly more likely to express willingness to participate compared to older pharmacists. Pharmacists aged ≤35 years were more than twice as likely to report willingness to participate compared to those aged >35 years (adjusted odds ratio [aOR] = 2.41, 95% CI: 1.42–4.09, *p* = 0.001).

Similarly, pharmacists with fewer years of professional experience demonstrated higher acceptance levels. Pharmacists with ≤10 years of experience were significantly more likely to report willingness compared to those with more than 10 years of experience (aOR = 1.96, 95% CI: 1.18–3.27, *p* = 0.009).

Employment setting was also significantly associated with acceptance. Pharmacists working in chain pharmacies were more likely to report willingness compared to those working in independent pharmacies (aOR = 2.17, 95% CI: 1.24–3.79, *p* = 0.006).

Familiarity with telemedicine concepts was a strong predictor of acceptance. Pharmacists who reported familiarity with telemedicine were significantly more likely to express willingness to participate (aOR = 2.88, 95% CI: 1.63–5.10, *p* < 0.001).

Additionally, pharmacists practicing in urban areas demonstrated higher acceptance compared to those practicing in rural or suburban areas (aOR = 1.74, 95% CI: 1.01–3.01, *p* = 0.046).

In contrast, higher perceived patient safety concern scores were significantly associated with lower likelihood of acceptance (aOR = 0.54, 95% CI: 0.38–0.76, *p* < 0.001), indicating that pharmacists with greater safety concerns were less likely to support telemedicine-enabled dispensing and delivery.

Gender was not significantly associated with acceptance (*p* = 0.317). Diagnostic checks did not indicate problematic multicollinearity among predictors, with all VIF values below 5 and tolerance values above 0.20. Sensitivity analyses excluding either age or years of professional experience, testing selected interaction terms, and exploring backward elimination yielded findings that were broadly consistent with the primary prespecified model.

Overall, these findings indicate that acceptance of telemedicine-enabled medication dispensing and delivery among community pharmacists in Jordan is influenced by demographic, professional, and perception-related factors, with younger age, fewer years of experience, chain pharmacy employment, urban practice, and greater familiarity with telemedicine associated with higher acceptance, while higher safety concerns were associated with resistance.

### 3.7. Qualitative Findings

A total of 22 semi-structured interviews were conducted with community pharmacists representing diverse demographic and professional backgrounds, including variation in years of experience, pharmacy ownership type, and geographic location. Thematic analysis identified five major themes related to pharmacists’ perceptions of telemedicine-enabled medication dispensing and delivery. Table 7 summarises the major themes and subthemes.

#### 3.7.1. Theme 1: Major Patient Safety Concerns

Nearly all participants expressed serious concerns regarding patient safety risks associated with remote dispensing and delivery. Pharmacists emphasized that direct interaction with patients is essential for identifying medication-related problems, verifying therapy appropriateness, and ensuring safe medication use.

Participants consistently described pharmacists as the final safety checkpoint in the medication use process.

“*We often identify prescribing errors only after speaking directly with the patient. Without this interaction, the risk of errors may increase*.”

(Participant 7, independent pharmacy, 12 years of experience)

Several participants also raised concerns regarding delivery conditions, particularly for temperature-sensitive medications.

“*Medications like insulin require strict storage conditions. Without proper control, effectiveness may be compromised*.”

(Participant 14, urban pharmacy, 9 years of experience)

#### 3.7.2. Theme 2: Unclear Professional Responsibility and Legal Liability

Uncertainty regarding legal and professional accountability emerged as a major concern. Pharmacists expressed reluctance to participate in systems where professional responsibilities and liability structures were not clearly defined.

“*If a medication error occurs, it is unclear who would be responsible. This uncertainty makes pharmacists hesitant*.”

(Participant 3, independent pharmacy, 15 years of experience)

Participants emphasized the importance of clear regulatory frameworks defining professional accountability.

#### 3.7.3. Theme 3: Lack of System Readiness and Infrastructure

Participants reported concerns regarding insufficient system readiness, including lack of integrated electronic prescription platforms, standardized protocols, and clear implementation procedures.

“*There is currently no unified electronic prescription system. Implementing this without proper infrastructure would be risky*.”

(Participant 11, chain pharmacy, 6 years of experience)

Pharmacists also expressed concerns regarding potential workflow disruption and increased operational complexity.

#### 3.7.4. Theme 4: Conditional Acceptance Based on Safeguards

Despite widespread concerns, some pharmacists expressed conditional acceptance if appropriate safeguards were implemented. Pharmacists emphasized that acceptance would depend on clear regulatory protections, pharmacist oversight, and robust safety protocols.

“*If the system ensures pharmacist verification, clear documentation, and legal protection, it could be implemented safely*.”

(Participant 19, urban pharmacy, 8 years of experience)

Younger pharmacists and those with prior exposure to digital health systems were more likely to express openness to future adoption.

#### 3.7.5. Theme 5: Proposed Solutions and Safeguards

Participants proposed several solutions to improve safety and feasibility of telemedicine-enabled medication dispensing and delivery.

Mandatory pharmacist verification of prescriptions was identified as a critical safeguard.

“*Pharmacists must remain responsible for reviewing and verifying prescriptions before dispensing*.”

(Participant 9, independent pharmacy, 11 years of experience)

Pharmacists also emphasized the importance of maintaining direct pharmacist–patient communication.

“*Direct communication with patients must remain part of the process, even if dispensing occurs remotely*.”

(Participant 16, chain pharmacy, 7 years of experience)

Participants recommended implementation of secure electronic prescription systems, standardized protocols, and clear legal protections.

“*Clear protocols and legal protections would significantly improve pharmacist confidence in the system*.”

(Participant 21, independent pharmacy, 18 years of experience)

Overall, pharmacists indicated that safe implementation would require robust regulatory frameworks, infrastructure development, and clearly defined professional responsibilities.

### 3.8. Integration of Quantitative and Qualitative Findings

Integration of quantitative and qualitative findings demonstrated strong convergence between datasets, with both approaches consistently indicating substantial resistance among community pharmacists toward telemedicine-enabled medication dispensing and delivery in its current form (Table 8).

Quantitatively, only 28.3% of pharmacists expressed willingness to participate, while 46.9% reported unwillingness and 24.9% indicated conditional acceptance. These findings were reinforced by qualitative interviews, in which pharmacists consistently expressed concerns regarding patient safety, legal liability, and system readiness. Qualitative data provided deeper insight into the underlying reasons for resistance, particularly pharmacists’ perception of their critical role as the final safeguard in medication safety and concerns that telemedicine-enabled dispensing could compromise this role.

Similarly, quantitative findings revealed very high levels of patient safety concern (mean score 4.4 ± 0.6), which were strongly supported by qualitative themes emphasizing risks related to reduced pharmacist–patient interaction, inability to verify patient conditions, and concerns regarding medication handling during delivery.

Quantitative regression analysis demonstrated that familiarity with telemedicine was associated with higher acceptance, while higher safety concern scores were associated with lower acceptance. These findings were further explained by qualitative data, which showed that pharmacists who were more familiar with telemedicine concepts were more open to future adoption, but emphasized the necessity of robust regulatory safeguards and clear professional accountability.

Additionally, quantitative findings identified lack of regulatory clarity and infrastructure as major implementation barriers, which were strongly corroborated by qualitative findings highlighting uncertainty regarding legal responsibility, absence of standardized protocols, and concerns regarding system readiness.

Overall, integration of quantitative and qualitative findings demonstrated consistent and complementary evidence indicating substantial resistance to telemedicine-enabled medication dispensing and delivery among community pharmacists in Jordan, primarily driven by patient safety concerns, legal uncertainty, and perceived lack of implementation readiness. However, both datasets indicated conditional acceptance if robust regulatory frameworks, safety protocols, and professional safeguards were established.

### 3.9. Perceived Facilitators for Safe Implementation of Telemedicine-Enabled Medication Dispensing and Delivery

Despite widespread concerns and resistance, pharmacists identified several facilitators that could improve acceptance and support the safe implementation of telemedicine-enabled medication dispensing and delivery systems.

As shown in Table 9, the most frequently identified facilitator was the requirement for mandatory pharmacist review and verification of all prescriptions prior to dispensing, supported by 94.6% (n = 331) of participants. Similarly, 93.4% (n = 327) emphasized the importance of establishing clear regulatory frameworks defining pharmacists’ roles, responsibilities, and legal protections.

A total of 91.1% (n = 319) of pharmacists reported that the implementation of standardized operating procedures (SOPs) governing electronic prescription verification, documentation, and pharmacist counseling would significantly improve their willingness to participate. Additionally, 90.3% (n = 316) emphasized the importance of ensuring direct pharmacist–patient communication, such as mandatory phone or video counseling prior to medication dispensing.

Delivery-specific safeguards were also identified as critical facilitators. Approximately 89.7% (n = 314) of pharmacists indicated that implementation of temperature-controlled delivery systems and standardized medication handling protocols would increase their confidence in telemedicine-enabled dispensing. Furthermore, 87.4% (n = 306) reported that the use of secure, integrated electronic prescription systems with clear audit trails would improve acceptance.

Legal and professional protection was also a key facilitator, with 92.0% (n = 322) indicating that clearly defined legal liability frameworks would increase their willingness to participate.

Overall, these findings indicate that while pharmacists currently express substantial resistance to telemedicine-enabled medication dispensing and delivery, acceptance may improve significantly if robust regulatory safeguards, pharmacist oversight, and infrastructure improvements are implemented.

## 4. Discussion

This convergent mixed-methods study provides one of the first policy-contextualised evaluations of Jordanian community pharmacists’ acceptance of the 2025 national instructions regulating telemedicine-enabled medication dispensing and delivery. Conducted during the critical early-implementation period (December 2025–February 2026), the findings reveal a substantial gap between regulatory authorisation and frontline professional readiness. Only 28.3% of pharmacists expressed immediate willingness to participate, 52.0% opposed national rollout, and 24.9% indicated conditional acceptance. Patient safety concerns were near-universal, implementation barriers were rated as severe, and the proposed safeguards received overwhelming endorsement. Qualitative themes reinforced these patterns by portraying pharmacists as the “final safety checkpoint” in the medication-use process. Taken together, resistance appears driven less by opposition to digital innovation itself than by perceived misalignment between regulatory design, system readiness, and the pharmacist’s clinical role.

A central interpretation of these findings is that Jordan’s experience reflects an implementation-readiness problem rather than simply an acceptance problem. The study was conducted at a very early stage following a governmental policy initiative, when legal authorization had been introduced but many of the organizational, professional, and infrastructural conditions required for safe implementation were still perceived as incomplete. This finding is consistent with broader implementation and socio-technical transition literature, which emphasizes that new technologies and service models rarely become embedded through formal policy decisions alone. Rather, implementation depends on stepwise alignment between regulation, infrastructure, workforce readiness, organizational routines, user trust, and professional legitimacy [10,11,12]. In this sense, the low acceptance observed in the present study should be interpreted as an indicator of insufficient implementation preparedness, rather than as simple resistance to telepharmacy or digital transformation.

A notable finding of this study is the clear distinction between conceptual acceptance of telepharmacy and resistance to its real-world perationalization under the 2025 regulatory framework. Earlier Jordanian studies reported moderate readiness toward telepharmacy as a general service model [17,18]. By contrast, the present findings—anchored in a live policy context—demonstrate that support at the conceptual level does not automatically translate into acceptance of a regulated, platform-mediated dispensing model that directly alters workflow, accountability, and pharmacist–patient interaction.

Patient safety emerged as the dominant organizing concern across both the quantitative and qualitative datasets. Pharmacists consistently expressed concern regarding reduced opportunities for direct patient assessment, limited ability to identify drug-related problems, increased risk of dispensing errors, and logistical vulnerabilities related to medication delivery and storage, particularly for temperature-sensitive therapies. These concerns are consistent with international evidence showing that telepharmacy models, when introduced without robust clinical integration and safeguards, may create new risks for medication safety and care quality [19,22]. Importantly, participants did not describe these risks as isolated operational inconveniences. Rather, they framed them as systemic vulnerabilities arising from fragmented communication, incomplete access to clinical information, and the absence of unified electronic health records. This interpretation is especially important because it indicates that safety concerns in this study were not merely reactive or emotional objections; they were grounded in pharmacists’ understanding of how medication safety depends on information continuity, clinical judgment, and effective communication at the point of dispensing.

These concerns are not unique to Jordan, but they appear more pronounced because the Jordanian model is being evaluated during an early and contested regulatory phase. International digital pharmacy guidance emphasizes that remote pharmacy services should preserve pharmacist involvement, patient safety, privacy, confidentiality, documentation quality, and appropriate medicine use [23]. In this context, telepharmacy is more likely to be acceptable when it is supported by clear legal authority, defined professional accountability, staff training, reliable communication systems, standardized documentation, quality assurance mechanisms, and direct pharmacist–patient communication. Compared with these best-practice principles, the Jordanian findings suggest that pharmacists’ resistance is mainly related to the perceived absence or immaturity of these enabling conditions, rather than rejection of remote pharmaceutical care itself.

The mixed-methods design is particularly valuable in interpreting this finding. The quantitative results established the scale and intensity of resistance, whereas the qualitative findings clarified that this resistance was rooted in concerns about safety, accountability, and professional role erosion rather than in simple technological conservatism. In other words, the survey data showed how widespread these concerns were, while the interviews explained why they were so strongly held. This complementary evidence strengthens the overall interpretation and supports the conclusion that the resistance documented in this study reflects a substantive professional judgment about implementation conditions rather than a superficial reluctance to adopt digital technologies.

The findings must also be Interpreted against the backdrop of Jordan’s 2025 regulatory instructions, which created a formal legal pathway for telemedicine-enabled dispensing and medication delivery. Although this regulatory step is important, the present findings suggest that legal authorization is not equivalent to implementation readiness. Pharmacists’ concerns point to several preparedness gaps that appear critical for successful implementation: regulatory clarity, professional responsibility, patient safety safeguards, digital infrastructure, workflow integration, staff training, and stakeholder engagement. These domains correspond closely to established technology implementation perspectives, including the technology–organization–environment framework, which emphasizes that adoption depends on technological capacity, organizational readiness, and external environmental conditions, and organizational readiness theory [10], which highlights the importance of shared commitment and perceived capability before change can be successfully implemented. The findings therefore suggest that the Jordanian framework has moved faster than the practical infrastructure and organizational conditions needed to support frontline adoption.

A major strength of this study Is the clear convergence between the empirical findings and the public position of the Jordan Pharmacists Association. The Association rejected the pharmacy-related provisions of the remote care framework on the grounds of insufficient stakeholder consultation, the absence of a unified national medical record system, and the risk of reducing pharmacists to a transactional or logistical role. These concerns were strongly echoed by participants in both the survey and the interviews. Pharmacists repeatedly described the model as one that could shift their role away from patient-centered care and toward task execution within a platform-based workflow. This alignment between profession-level advocacy and frontline pharmacist perspectives strengthens the credibility of the findings and suggests that the observed resistance is not merely individual or anecdotal but reflects a wider systemic concern within the profession. More importantly, it indicates that the implementation challenge is not simply technical, but institutional and relational, involving trust, legitimacy, and meaningful professional engagement in policy design.

Another major theme arising from the data concerns professional identity. Participants expressed concern that the current model could diminish opportunities for meaningful patient counseling, therapeutic monitoring, and professional judgment, thereby redefining the pharmacist’s role in a more logistical or commercial direction. This concern is well aligned with the wider digital health literature, which cautions that poorly integrated technological systems can unintentionally marginalize healthcare professionals if their core clinical contributions are not actively preserved within implementation design [7,10]. In the present study, this issue was not peripheral. Rather, it appears to function as one of the mechanisms through which safety concerns, liability concerns, and implementation resistance became intertwined. The pharmacists’ objections therefore seem to reflect not only concerns about operational burden, but also a deeper concern that a poorly designed remote dispensing model could compromise the profession’s clinical identity.

The multivariable findings provide further Insight Into Ih groups may be more open to future adoption. Willingness to participate was significantly higher among younger pharmacists, those with fewer years of experience, pharmacists working in chain pharmacies, those practicing in urban areas, and those already familiar with telemedicine concepts. By contrast, higher safety concern scores were strongly associated with lower willingness. The qualitative findings help explain these associations. Older and more experienced pharmacists, particularly those working in independent pharmacies, articulated deeper concerns regarding workflow disruption, legal exposure, and erosion of professional value. Younger pharmacists and those more familiar with digital health were not uncritically supportive, but were more likely to express conditional openness provided that robust safeguards were in place. This is one of the clearest analytic strengths of the study: the quantitative analysis identifies the predictors of willingness, while the qualitative analysis explains the mechanisms underlying them. The implication for policymakers is that resistance is not uniform across the sector and that implementation strategies should therefore be tailored rather than generic.

Despite the overall resistance, one of the most constructive findings of this study is that opposition was directed not toward telepharmacy as a concept, but toward its current implementation model. Pharmacists identified a coherent set of conditions under which acceptance could improve substantially. These included integrated electronic health records, mandatory pharmacist verification of prescriptions, direct phone or video counseling, standardized operating procedures, temperature-controlled delivery protocols, and explicit legal protections with indemnity. These facilitators received near-unanimous endorsement and suggest that pharmacists are not rejecting innovation itself. Rather, they are asking for a model that preserves safety, accountability, and professional integrity. This interpretation is consistent with implementation experiences in other settings, where co-designed safeguards, targeted training, and iterative regulatory refinement were necessary to overcome early skepticism and translate telepharmacy from concept into acceptable practice [17]. The present study therefore shifts the conversation from whether pharmacists support telepharmacy in principle to what conditions are necessary for them to support it in practice.

From a policy perspective, the findings suggest that implementation should proceed through a prioritized, safeguard-centred roadmap rather than immediate broad rollout. First, regulatory refinement should clarify the pharmacist’s professional authority, minimum verification duties, documentation requirements, liability boundaries, responsibility for delivery-related incidents, and exclusion criteria for medicines unsuitable for remote dispensing. Second, patient safety safeguards should be made mandatory, including pharmacist verification before dispensing, direct pharmacist–patient counselling by phone or video, standardized electronic prescription review, drug-interaction and contraindication screening, temperature-controlled delivery procedures, identity verification at handover, and auditable documentation of each step. Third, digital infrastructure should be strengthened before scale-up through secure integrated electronic prescription systems, audit trails, access to relevant medication history where possible, and reliable communication channels between prescribers, pharmacists, patients, and delivery providers. Fourth, workflow integration and staff training should be addressed through practical SOPs, pharmacist training modules, simulated dispensing scenarios, and clear escalation pathways for incomplete prescriptions, counselling failures, storage breaches, or suspected medication errors. Fifth, stakeholder engagement should be institutionalized through structured dialogue between the Ministry of Health, the Jordan Pharmacists Association, community pharmacists, physicians, digital platform providers, delivery companies, insurers, and patient representatives. Finally, implementation should begin with phased pilot programs in settings with higher readiness, such as selected urban and chain pharmacies, while ensuring that independent and rural pharmacies are included in later phases to avoid widening service inequities.

A logical next step would be a longitudinal follow-up study after one to two years of implementation or pilot testing. Such a study should assess whether pharmacists’ acceptance changes as regulations become clearer, infrastructure improves, and pharmacists gain practical experience with the model. It should also move beyond stated willingness to evaluate implementation outcomes, including uptake, workflow integration, counselling quality, documentation completeness, medication delivery incidents, patient safety events, patient satisfaction, pharmacist workload, and equity of access across urban, rural, independent, and chain pharmacy settings. A follow-up mixed-methods or implementation-effectiveness design would help identify which safeguards contribute most meaningfully to safer and more acceptable telemedicine-enabled dispensing practice in Jordan.

## 5. Conclusions

In conclusion, Jordanian community pharmacists express substantial yet principled resistance to the current model of telemedicine-enabled medication dispensing. Their concerns centre on patient safety, professional accountability, and preservation of their clinical role rather than opposition to digital innovation. By linking quantitative predictors, qualitative mechanisms, and strongly endorsed safeguards to concrete policy recommendations, this study offers a pragmatic roadmap for translating regulatory intent into safe, acceptable, and equitable practice. A collaborative, safeguard-centred approach is essential If telepharmacy Is to be Iy Integrated Into Jordan’s healthcare system. Future longitudinal and multi-stakeholder studies should evaluate whether evidence-based refinements improve professional acceptance, patient safety, medication adherence, and healthcare equity.

## 6. Limitations

This study has several limitations that should be acknowledged. The cross-sectional quantitative component precludes causal inference, and data were collected during an active period of regulatory debate, which may have influenced responses. In addition, the study assessed pharmacists’ perceptions and stated willingness rather than actual participation in an implemented telemedicine-enabled dispensing system. Therefore, it was not designed to evaluate long-term outcomes such as changes in acceptance over time, actual implementation success, patient safety incidents, medication error rates, or sustained workflow changes after implementation. Although qualitative saturation was achieved, social desirability bias remains possible in a policy-sensitive environment. Because the study was conducted during early regulatory rollout, perceptions may evolve as implementation becomes more familiar or as supporting systems are developed. In addition, the focus on community pharmacists, while appropriate to the study aim, means that other relevant perspectives, including those of physicians, patients, regulators, and delivery providers, were not captured. Future longitudinal, implementation-based, and multi-stakeholder studies are therefore needed to evaluate whether regulatory refinements and safety safeguards improve professional acceptance, patient safety, workflow integration, and healthcare equity over time.

## Figures and Tables

**Table 1:** Demographic and professional characteristics of participating pharmacists (n = 350).

Characteristic	Category	n	%
Gender	Male	144	41.1
	Female	206	58.9
Age group	22–25	48	13.7
	26–35	197	56.3
	36–45	72	20.6
	>45	33	9.4
Years of experience	<5 years	118	33.7
	5–10 years	97	27.7
	10–20 years	97	27.7
	>20 years	38	10.9
Pharmacy type	Independent	254	72.6
	Chain pharmacy	96	27.4
Practice location	Urban	277	79.1
	Rural/Suburban	73	20.9
Daily prescriptions dispensed	<50	113	32.3
	50–100	154	44.0
	>100	83	23.7
General Familiarity with telemedicine	Yes	222	63.4
	No	128	36.6
Experience handling electronically received prescriptions	Yes	146	41.7
	No	204	58.3
Awareness of recent regulatory/policy discussions on telemedicine-enabled dispensing	Yes	122	34.9
	No	228	65.1

**Table 2:** Awareness and knowledge of telemedicine-enabled dispensing and delivery (n = 350).

Variable	Category	n	%
Awareness of telemedicine-enabled medication dispensing/delivery as specific model	Aware	216	61.7
	Not aware	134	38.3
Self-reported level of understanding	Good understanding	65	18.6
	Basic understanding	151	43.1
	Poor/no understanding	134	38.3
Awareness of specific formal regulatory/policy initiatives in Jordan	Yes	103	29.4
	No/uncertain	247	70.6
Sources of information	Colleagues	164	46.9
	Social media/online	147	42.0
	Professional organizations	100	28.6
	Continuing education	55	15.7
	Formal training	45	12.9

Note: The awareness-related variables in Table 1 and Table 2 were intentionally assessed as distinct constructs. Table 1 reports background characteristics, including general familiarity with telemedicine and general awareness of recent regulatory or policy discussions. Table 2 reports model-specific awareness, including awareness of telemedicine-enabled medication dispensing/delivery as a specific service model, self-reported operational understanding, sources of information, and awareness of specific formal regulatory or policy initiatives in Jordan. Therefore, differences in percentages across awareness variables reflect differences in the constructs measured rather than inconsistency in the data.

**Table 3:** Acceptance and willingness to adopt telemedicine-enabled dispensing and delivery under current conditions and at the national implementation level (n = 350).

Outcome Domain	Category	n	%
Panel A: Willingness to participate under current conditions	Yes	99	28.3
	No	164	46.9
	Conditional	87	24.9
Panel B: Position regarding national implementation in its current form	Support	57	16.3
	Neutral/uncertain	111	31.7
	Oppose	182	52.0

Note: Panel A reflects pharmacists’ personal willingness to participate in telemedicine-enabled dispensing under current conditions. Panel B reflects their broader position regarding national implementation of the model in its current form. These are separate outcomes and should not be interpreted as subcategories of a single variable.

**Table 4:** Perceived patient safety risks (n = 350).

Safety Concern	Agree/Strongly Agree n (%)
Inability to adequately assess patient condition remotely	320 (91.4%)
Increased risk of drug interactions or inappropriate therapy	312 (89.1%)
Increased risk of medication errors	311 (88.9%)
Reduced effectiveness of pharmacist–patient counseling	303 (86.6%)
Unclear legal and professional responsibility	297 (84.9%)
Improper storage conditions during delivery	288 (82.3%)
Incomplete or unclear electronic prescription information	278 (79.4%)

**Table 5:** Perceived implementation barriers (n = 350).

Implementation Barrier	Agree/Strongly Agree n (%)
Lack of clear regulatory framework	322 (92.0%)
Unclear legal and professional liability	314 (89.7%)
Absence of standardized dispensing protocols	306 (87.4%)
Insufficient technological infrastructure	296 (84.6%)
Lack of standardized medication delivery protocols	287 (82.0%)
Potential disruption to pharmacy workflow	279 (79.7%)
Increased administrative burden	269 (76.9%)
Potential negative economic impact on pharmacies	257 (73.4%)

Note: Mean overall barrier severity score: 4.5 ± 0.5 (on a 5-point Likert scale).

**Table 6:** Multivariable logistic regression analysis of factors associated with willingness to participate (n = 350).

Variable	Adjusted Odds Ratio (aOR)	95% CI	*p*-Value
Age ≤ 35 years	2.41	1.42–4.09	0.001
Experience ≤ 10 years	1.96	1.18–3.27	0.009
Chain pharmacy employment	2.17	1.24–3.79	0.006
Urban practice location	1.74	1.01–3.01	0.046
Familiarity with telemedicine	2.88	1.63–5.10	<0.001
Higher safety concern score	0.54	0.38–0.76	<0.001
Female gender	1.21	0.83–1.77	0.317

Note: Model statistics: Nagelkerke R^2^ = 0.29, Hosmer–Lemeshow test *p* = 0.61, Overall model accuracy = 72.3%.

**Table 7:** Qualitative themes and subthemes.

Theme	Subthemes
Patient safety concerns	Risk of medication errors, inability to assess patients, delivery storage risks, reduced counseling effectiveness
Legal and professional concerns	Unclear liability, lack of accountability frameworks
System readiness and infrastructure	Lack of integrated systems, absence of protocols, workflow disruption
Conditional acceptance	Acceptance dependent on regulatory safeguards, pharmacist oversight
Proposed solutions and safeguards	Pharmacist verification, direct pharmacist–patient communication, standardized protocols, legal protection

**Table 8:** Joint display integrating quantitative and qualitative findings.

Domain	Quantitative Findings	Qualitative Findings	Integrated Interpretation
Acceptance and willingness	Only 28.3% willing to participate; 46.9% unwilling; 24.9% conditional acceptance	Pharmacists expressed strong resistance but conditional openness if safeguards exist	Low acceptance is driven by safety and regulatory concerns; willingness depends on presence of safeguards
Patient safety concerns	91.4% concerned about inability to assess patients remotely; 88.9% concerned about medication errors	Pharmacists described themselves as the “final safety checkpoint” and feared losing ability to detect prescribing errors	High safety concern reflects perceived erosion of pharmacists’ clinical safety role in telemedicine-enabled workflows
Legal and professional accountability	89.7% reported unclear legal responsibility as major barrier	Pharmacists expressed uncertainty about liability and reluctance to assume responsibility without clear legal protection	Legal ambiguity is a primary driver of resistance and a key barrier to implementation
Infrastructure readiness	84.6% reported insufficient technological infrastructure	Pharmacists reported lack of integrated electronic systems and standardized protocols	Perceived infrastructure gaps contribute to lack of confidence in safe implementation
Workflow and operational impact	79.7% reported potential workflow disruption	Pharmacists expressed concern about changes to traditional workflow and increased complexity	Workflow disruption contributes to resistance and uncertainty regarding system feasibility
Delivery-related safety concerns	82.3% concerned about improper storage during delivery	Pharmacists emphasized risks to temperature-sensitive medications during transport	Delivery introduces new safety risks not present in traditional dispensing models
Predictors of acceptance	Younger pharmacists more accepting (aOR 2.41); familiarity with telemedicine increased acceptance (aOR 2.88)	Younger pharmacists and those with digital exposure expressed greater openness to future adoption	Familiarity with digital health increases acceptance, suggesting experience reduces perceived risk
Conditional acceptance factors	24.9% conditionally willing to participate	Pharmacists emphasized need for regulatory clarity, pharmacist oversight, and safety protocols	Acceptance is contingent upon regulatory safeguards and clear professional roles

**Table 9:** Perceived facilitators for safe implementation (n = 350).

Facilitator	Agree/Strongly Agree n (%)
Mandatory pharmacist prescription verification	331 (94.6%)
Clear regulatory framework and legal protections	327 (93.4%)
Clearly defined legal liability structure	322 (92.0%)
Standardized operating procedures (SOPs)	319 (91.1%)
Mandatory pharmacist–patient counseling prior to dispensing	316 (90.3%)
Temperature-controlled medication delivery systems	314 (89.7%)
Integrated electronic prescription system with audit trail	306 (87.4%)

Note: Mean facilitator agreement score: 4.7 ± 0.4.

## Data Availability

The data supporting the findings of this study are available from the corresponding author upon reasonable request. Due to the qualitative nature of part of the study and the need to protect participant confidentiality, interview transcripts and other potentially identifiable data are not publicly available.

## Supplementary Materials

The following supporting information can be downloaded at: https://www.mdpi.com/article/10.3390/healthcare14101346/s1.

## Abbreviations

The following abbreviations are used in this manuscript:

COREQ	Consolidated Criteria for Reporting Qualitative Research
COVID-19	Coronavirus Disease 2019
GRAMMS	Good Reporting of A Mixed Methods Study
SOPs	standard operating procedures
STROBE	Strengthening the Reporting of Observational Studies in Epidemiology
