# Supplementary material for: Community Pharmacists’ Acceptance of Telemedicine-Enabled Medication Dispensing in Jordan: A Mixed-Methods Study of Patient Safety Concerns, Implementation Barriers, and Required Safeguards
Source: Healthcare (Basel). 2026 May 14;14(10):1346. doi: 10.3390/healthcare14101346 (PMC13206089; doi:10.3390/healthcare14101346)
Supplement: Supplementary file 1 [file healthcare-14-01346-s001.zip › Coding Tree (Supplementary S4).docx]

**Coding Tree**

*Community Pharmacists’ Acceptance of Telemedicine-Enabled Medication Dispensing in Jordan*

**Supplementary Material S4. Summary coding tree of themes and subthemes.**

| **Theme** | **Subthemes / codes** |
| --- | --- |
| **Theme 1. Major patient safety concerns** | - Risk of medication errors in remote dispensing workflows - Inability to adequately assess patients without direct interaction - Reduced ability to detect inappropriate therapy, contraindications, and drug interactions - Reduced effectiveness of pharmacist–patient counseling - Delivery-related safety risks   - improper storage conditions   - transport of temperature-sensitive medications   - traceability and recipient verification concerns |
| **Theme 2. Legal and professional concerns** | - Unclear legal liability if medication errors occur - Lack of clear accountability frameworks - Reluctance to participate without explicit legal protection - Concern about unfair transfer of responsibility to pharmacists - Need for clearly defined professional roles within telemedicine-enabled dispensing |
| **Theme 3. Lack of system readiness and infrastructure** | - Lack of integrated electronic prescription systems - Absence of a unified medical record or medication tracking system - Lack of standardized dispensing and delivery protocols - Insufficient technological infrastructure - Workflow disruption and increased operational complexity - Concerns about implementation feasibility in routine practice |
| **Theme 4. Conditional acceptance based on safeguards** | - Acceptance dependent on regulatory safeguards - Acceptance dependent on pharmacist oversight - Acceptance dependent on clear documentation procedures - Acceptance dependent on legal clarity and professional protection - Greater openness among younger pharmacists and those familiar with digital health |
| **Theme 5. Proposed solutions and safeguards** | - Mandatory pharmacist verification of prescriptions before dispensing - Direct pharmacist–patient communication maintained within the process - Standardized operating procedures for prescription verification, counseling, dispensing, and delivery - Secure integrated electronic prescription systems with audit trails - Clear legal protections and defined liability structures - Temperature-controlled delivery systems and medication-handling safeguards -  Phased implementation supported by infrastructure development and professional engagement |
