# Supplementary material for: Community Pharmacists’ Acceptance of Telemedicine-Enabled Medication Dispensing in Jordan: A Mixed-Methods Study of Patient Safety Concerns, Implementation Barriers, and Required Safeguards
Source: Healthcare (Basel). 2026 May 14;14(10):1346. doi: 10.3390/healthcare14101346 (PMC13206089; doi:10.3390/healthcare14101346)
Supplement: Supplementary file 1 [file healthcare-14-01346-s001.zip › COREQ checklist telemedicine dispensing_Jordan-2 (Supplementary S1).docx]

**COREQ Checklist (Revised Version)**

*Community Pharmacists’ Acceptance of Telemedicine-Enabled Medication Dispensing in Jordan: A Mixed-Methods Study of Patient Safety Concerns, Implementation Barriers, and Required Safeguards*

| **Domain** | **Item** | **Guide questions / description** | **Page No.** | **How addressed in the manuscript** |
| --- | --- | --- | --- | --- |
| **Domain 1: Research team and reflexivity** | | | | |
| Personal characteristics | 1. Interviewer/facilitator | Which author/s conducted the interview or focus group? | 8 | A male researcher with a background in pharmacy practice and health services research conducted all interviews. |
| Personal characteristics | 2. Credentials | What were the researcher’s credentials? E.g., PhD, MD | 8 | The interviewer is described as an academic faculty member with a PhD degree and prior qualitative interviewing experience. |
| Personal characteristics | 3. Occupation | What was their occupation at the time of the study? | 8 | The interviewer was an academic researcher/faculty member in pharmacy practice. |
| Personal characteristics | 4. Gender | Was the researcher male or female? | 8 | The interviewer is explicitly identified as male. |
| Personal characteristics | 5. Experience and training | What experience or training did the researcher have? | 8 | Prior training and experience in qualitative interviewing are reported. |
| Relationship with participants | 6. Relationship established | Was a relationship established prior to study commencement? | 8 | No prior personal or professional relationship existed between the interviewer and participants. |
| Relationship with participants | 7. Participant knowledge of the interviewer | What did the participants know about the researcher? e.g., personal goals, reasons for doing the research | 8 | Participants were told that the interviewer was an academic researcher in pharmacy practice, that the study was for research purposes only, and that he was not affiliated with regulators or enforcement bodies. |
| Relationship with participants | 8. Interviewer characteristics | What characteristics were reported about the interviewer/facilitator? e.g., bias, assumptions, reasons and interests in the research topic | 8 | Reflexivity is addressed by acknowledging the research team’s professional background and the possibility of privileging medication safety and professional autonomy concerns; mitigation strategies are described. |
| **Domain 2: Study design** | | | | |
| Theoretical framework | 9. Methodological orientation and theory | What methodological orientation was stated to underpin the study? e.g., grounded theory, discourse analysis, ethnography, phenomenology, content analysis | 7-8 | A descriptive qualitative approach with reflexive thematic analysis following Braun and Clarke and a hybrid inductive-deductive framework is reported. |
| Participant selection | 10. Sampling | How were participants selected? e.g., purposive, convenience, consecutive, snowball | 7 | Purposive sampling with maximum variation was used. |
| Participant selection | 11. Method of approach | How were participants approached? e.g., face-to-face, telephone, mail, email | 7 | Potential interview participants were approached by phone, email, or direct message through professional networks, and some were invited through survey follow-up contact. |
| Participant selection | 12. Sample size | How many participants were in the study? | 7, 20 | Twenty-two pharmacists completed interviews; this is also restated in Results. |
| Participant selection | 13. Non-participation | How many people refused to participate or dropped out? Reasons? | 7 | Thirty pharmacists were invited, 22 completed interviews, and 8 declined mainly because of time constraints. |
| Setting | 14. Setting of data collection | Where was the data collected? e.g., home, clinic, workplace | 8 | Interviews were conducted individually in private settings, either online through secure platforms or in person, according to participant preference. |
| Setting | 15. Presence of non-participants | Was anyone else present besides the participants and researchers? | 8 | No non-participants were present. |
| Setting | 16. Description of sample | What are the important characteristics of the sample? e.g., demographic data, date | 7, 20 | The interview sample is described as varying by sex, years of experience, pharmacy type, practice location, and level of acceptance; broader pharmacist characteristics are reported in Results Table 1. |
| Data collection | 17. Interview guide | Were questions, prompts, guides provided by the authors? Was it pilot tested? | 7 | A semi-structured interview guide is reported in Supplementary Material S3; it was reviewed by experts and piloted with three community pharmacists. |
| Data collection | 18. Repeat interviews | Were repeat interviews carried out? If yes, how many? | 7 | No repeat interviews were conducted. |
| Data collection | 19. Audio/visual recording | Did the research use audio or visual recording to collect the data? | 8 | All interviews were audio-recorded with written or verbal consent. |
| Data collection | 20. Field notes | Were field notes made during and/or after the interview or focus group? | 8 | Field notes were taken during and immediately after each interview. |
| Data collection | 21. Duration | What was the duration of the interviews or focus group? | 8 | Interviews lasted approximately 30-45 minutes. |
| Data collection | 22. Data saturation | Was data saturation discussed? | 7 | Recruitment continued until thematic saturation was reached, defined as the point at which no substantively new themes or insights emerged. |
| Data collection | 23. Transcripts returned | Were transcripts returned to participants for comment and/or correction? | 8 | Full transcripts were not returned; instead, selected participants reviewed concise summaries of emerging themes for interpretive accuracy. |
| Data analysis | 24. Number of data coders | How many data coders coded the data? | 8 | Two researchers independently coded an initial subset of transcripts; the remaining transcripts were coded by the primary researcher after framework stabilization. |
| Data analysis | 25. Description of the coding tree | Did authors provide a description of the coding tree? | 8; Supplementary Material S4 | A structured coding framework with higher-order themes and subthemes is described, and a summary coding tree is reported in Supplementary Material S4. |
| Data analysis | 26. Derivation of themes | Were themes identified in advance or derived from the data? | 8 | Themes were generated using a hybrid inductive-deductive approach: deductive sensitising concepts informed analysis while inductive coding allowed Jordan-specific issues to emerge. |
| Data analysis | 27. Software | What software, if applicable, was used to manage the data? | 8 | NVivo Release 14 was used for coding and data management. |
| Data analysis | 28. Participant checking | Did participants provide feedback on the findings? | 8 | Yes, selected participants reviewed concise summaries of emerging themes. |
| **Domain 3: Analysis and findings** | | | | |
| Reporting | 29. Quotations presented | Were participant quotations presented to illustrate the themes/findings? Was each quotation identified? | 20-22 | Illustrative quotations are provided in Results and identified by participant number and brief context (e.g., pharmacy type and years of experience). |
| Reporting | 30. Data and findings consistent | Was there consistency between the data presented and the findings? | 20-23 | Yes. The themes, quotations, and integrated interpretation are aligned and mutually reinforcing. |
| Reporting | 31. Clarity of major themes | Were major themes clearly presented in the findings? | 20-23 | Yes. Five major themes are clearly presented in the qualitative findings, with a summary table of themes and subthemes. |
| Reporting | 32. Clarity of minor themes | Is there a description of diverse cases or discussion of minor themes? | 20-23 | The manuscript presents convergence, conditional acceptance, and nuanced differences in openness across participant groups, although minor/disconfirming cases are described briefly rather than as separate named minor themes. |
