# Supplementary material for: Community Pharmacists’ Acceptance of Telemedicine-Enabled Medication Dispensing in Jordan: A Mixed-Methods Study of Patient Safety Concerns, Implementation Barriers, and Required Safeguards
Source: Healthcare (Basel). 2026 May 14;14(10):1346. doi: 10.3390/healthcare14101346 (PMC13206089; doi:10.3390/healthcare14101346)
Supplement: Supplementary file 1 [file healthcare-14-01346-s001.zip › Interview Guide (Supplementary Material S3).docx]

**Semi-Structured Interview Guide**

*Community Pharmacists’ Acceptance of Telemedicine-Enabled Medication Dispensing in Jordan*

**Supplementary Material S3: Interview Guide**

| **Purpose of the guide** |
| --- |
| This guide was developed to explore community pharmacists’ perceptions of telemedicine-enabled medication dispensing and delivery in Jordan, with particular attention to patient safety, legal and professional responsibility, infrastructure readiness, workflow implications, and conditions required for acceptable implementation. |

**Interviewer instructions**

- Use the guide flexibly. Questions may be reordered depending on participant flow and emerging issues.
- Begin with broad, open-ended questions, then use probes only when clarification, examples, or deeper explanation are needed.
- Maintain a neutral, non-leading tone throughout the interview.
- Encourage participants to speak from their own practice experience and perceptions.
- Record brief field notes during and immediately after the interview.

**Opening script**

- Thank you for agreeing to take part in this interview.
- We are conducting this study to understand community pharmacists’ views on telemedicine-enabled medication dispensing and delivery in Jordan.
- Your participation is voluntary. There are no right or wrong answers; we are interested in your honest opinions and experiences.
- The interview will take approximately 30–45 minutes.
- With your permission, the interview will be audio-recorded to ensure accuracy.
- Your responses will be kept confidential, de-identified, and used only for research purposes.
- You may decline to answer any question or stop the interview at any time.

**Participant profile sheet**

| **Participant code** |  |
| --- | --- |
| **Interview date** |  |
| **Mode of interview** | Online / In person |
| **Pharmacy type** | Independent / Chain |
| **Practice location** | Urban / Rural |
| **Years of experience** |  |
| **Sex** | Male / Female |
| **Level of acceptance toward telemedicine-enabled dispensing** | Supportive / Opposed / Conditional acceptance |

**Section A. General understanding and overall views**

**1. How would you describe your understanding of telemedicine-enabled medication dispensing and delivery in the Jordanian context?**

*Optional probes:*

- What comes to mind when you hear this model?
- How do you think it differs from traditional community pharmacy dispensing?

**2. What is your overall opinion about implementing this model in Jordan?**

*Optional probes:*

- Do you generally support it, oppose it, or see it as conditionally acceptable?
- Why do you feel that way?

**Section B. Patient safety concerns**

**3. From a patient safety perspective, what concerns do you think may arise if medicines are dispensed and delivered through telemedicine-linked systems?**

*Optional probes:*

- Are you concerned about medication errors, inappropriate therapy, or missed drug-related problems?
- How might reduced direct interaction with patients affect safe dispensing?

**4. In your view, how important is face-to-face pharmacist–patient interaction in preventing medication-related problems?**

*Optional probes:*

- Can you give examples of situations where speaking directly with a patient changes your decision or advice?
- What might be lost if this interaction becomes remote?

**5. Are there any particular categories of medicines that you believe should not be dispensed through remote systems?**

*Optional probes:*

- Why would these medicines be higher risk?
- Would storage, monitoring, counselling, or misuse risks affect your view?

**6. What concerns, if any, do you have about medication delivery itself?**

*Optional probes:*

- Storage conditions?
- Temperature-sensitive products?
- Correct recipient identification?
- Traceability during transport?

**Section C. Legal, ethical, and professional responsibility**

**7. What do you think about pharmacists’ professional responsibility within telemedicine-enabled dispensing systems?**

*Optional probes:*

- Do you think the pharmacist remains the final safety checkpoint?
- Would this model change your professional role?

**8. If a medication error occurs in a remote dispensing pathway, who do you think should be responsible?**

*Optional probes:*

- The prescribing physician?
- The pharmacist?
- The platform?
- The delivery provider?
- Shared responsibility?

**9. Do you feel that current regulations provide enough legal clarity and protection for pharmacists?**

*Optional probes:*

- What is still unclear?
- What legal or regulatory changes would improve confidence?

**Section D. System readiness and workflow feasibility**

**10. How ready do you think the current healthcare and pharmacy system in Jordan is for this model?**

*Optional probes:*

- Electronic prescriptions?
- Unified medical records?
- Audit trails?
- Communication systems?

**11. How might this model affect daily workflow in community pharmacies?**

*Optional probes:*

- Would it increase administrative burden?
- Would it affect staffing, counselling time, or verification procedures?
- Would the impact differ between independent and chain pharmacies?

**12. Do you think implementation challenges would differ by setting?**

*Optional probes:*

- Urban versus rural areas?
- Small independent pharmacies versus chain pharmacies?
- Why?

**Section E. Conditions for acceptance and required safeguards**

**13. Under what conditions, if any, would you be willing to accept telemedicine-enabled medication dispensing and delivery?**

*Optional probes:*

- What safeguards would need to be in place first?
- What would make you feel more confident in the system?

**14. What specific safeguards do you believe are essential for safe implementation?**

*Optional probes:*

- Mandatory pharmacist prescription verification?
- Direct pharmacist–patient counselling by phone or video?
- Standard operating procedures?
- Secure electronic systems with audit trails?
- Temperature-controlled delivery protocols?

**15. What role should pharmacists have in the design and regulation of this model?**

*Optional probes:*

- Should pharmacists be involved in developing protocols or implementation guidance?
- How can policymakers better involve the profession?

**Section F. Final reflections**

**16. In your opinion, what is the single most important issue that policymakers must address before implementing this model more widely?**

*Optional probes:*

- Why is this the top priority?

**17. Is there anything else you would like to add about telemedicine-enabled dispensing and delivery that we have not discussed?**

**Closing statement**

- Thank you very much for your time and for sharing your views.
- Your input is valuable for understanding how telemedicine-enabled dispensing can be implemented safely and acceptably in Jordan.
- If needed, we may contact you again only for clarification or member checking of summary findings.
