# Supplementary material for: Community Pharmacists’ Acceptance of Telemedicine-Enabled Medication Dispensing in Jordan: A Mixed-Methods Study of Patient Safety Concerns, Implementation Barriers, and Required Safeguards
Source: Healthcare (Basel). 2026 May 14;14(10):1346. doi: 10.3390/healthcare14101346 (PMC13206089; doi:10.3390/healthcare14101346)
Supplement: Supplementary file 1 [file healthcare-14-01346-s001.zip › Questionnaire (Supplementary Material S2).docx]

**Questionnaire**

*Community Pharmacists’ Acceptance of Telemedicine-Enabled Medication Dispensing in Jordan*

**Supplementary Material S2**

| **Instructions to participants** |
| --- |
| This questionnaire explores community pharmacists’ views on telemedicine-enabled medication dispensing and delivery in Jordan. Please answer all items based on your current understanding and professional opinion. There are no right or wrong answers. All responses are confidential and will be used for research purposes only. |

**Response format**

**For Likert-scale items, please use the following scale:** 1 = Strongly disagree, 2 = Disagree, 3 = Neutral/Unsure, 4 = Agree, 5 = Strongly agree.

**Section A. Demographic and professional characteristics**

| **Item** | **Response** |
| --- | --- |
| 1. Gender | ☐ Male ☐ Female |
| 2. Age group | ☐ 22–25 ☐ 26–35 ☐ 36–45 ☐ >45 |
| 3. Years of professional experience | ☐ <5 years ☐ 5–10 years ☐ 10–20 years ☐ >20 years |
| 4. Pharmacy type | ☐ Independent community pharmacy ☐ Chain pharmacy |
| 5. Practice location | ☐ Urban ☐ Rural/Suburban |
| 6. Average number of prescriptions dispensed daily | ☐ <50 ☐ 50–100 ☐ >100 |
| 7. Familiarity with the concept of telemedicine | ☐ Yes ☐ No |
| 8. Prior experience handling electronically received prescriptions | ☐ Yes ☐ No |
| 9. Awareness of recent regulatory or policy discussions in Jordan regarding telemedicine-enabled dispensing | ☐ Yes ☐ No |

**Section B. Awareness, understanding, and acceptance**

| **Item** | **Response** |
| --- | --- |
| 10. Are you aware of the concept of telemedicine-enabled medication dispensing and delivery? | ☐ Yes ☐ No |
| 11. How would you rate your understanding of how such systems operate? | ☐ Good understanding ☐ Basic understanding ☐ Poor / no understanding |
| 12. Main sources of information about this model (select all that apply) | ☐ Colleagues ☐ Social media / online discussions ☐ Professional organizations / regulatory announcements ☐ Continuing education ☐ Formal training |
| 13. Are you aware of specific regulatory or policy initiatives in Jordan related to telemedicine-enabled medication dispensing and delivery? | ☐ Yes ☐ No / Unsure |
| 14. Would you be willing to participate in telemedicine-enabled medication dispensing and delivery under current conditions? | ☐ Yes ☐ No ☐ Conditional acceptance |
| 15. What is your position regarding national implementation of this model in its current form? | ☐ Support ☐ Neutral / uncertain ☐ Oppose |

**Section C. Perceived patient safety risks**

| **Item** | **1** | **2** | **3** | **4** | **5** |
| --- | --- | --- | --- | --- | --- |
| 16. Remote dispensing may reduce my ability to adequately assess the patient’s condition before dispensing. | ☐ | ☐ | ☐ | ☐ | ☐ |
| 17. Remote dispensing may reduce my ability to identify potential drug–drug interactions, contraindications, or inappropriate doses. | ☐ | ☐ | ☐ | ☐ | ☐ |
| 18. Telemedicine-enabled dispensing may increase the risk of medication errors if implemented without proper safeguards. | ☐ | ☐ | ☐ | ☐ | ☐ |
| 19. Reduced direct pharmacist–patient interaction may negatively affect patient counselling and medication safety. | ☐ | ☐ | ☐ | ☐ | ☐ |
| 20. Legal and professional accountability may become unclear if medication errors occur within remote dispensing workflows. | ☐ | ☐ | ☐ | ☐ | ☐ |
| 21. Medication delivery may expose medicines to improper storage conditions during transport. | ☐ | ☐ | ☐ | ☐ | ☐ |
| 22. Electronic prescriptions used in remote dispensing may sometimes be incomplete or unclear. | ☐ | ☐ | ☐ | ☐ | ☐ |
| 23. Overall, telemedicine-enabled medication dispensing and delivery poses important patient safety risks. | ☐ | ☐ | ☐ | ☐ | ☐ |

**Scale:** 1 = Strongly disagree, 2 = Disagree, 3 = Neutral/Unsure, 4 = Agree, 5 = Strongly agree.

**Section D. Perceived implementation barriers**

| **Item** | **1** | **2** | **3** | **4** | **5** |
| --- | --- | --- | --- | --- | --- |
| 24. There is a lack of a clear regulatory framework defining pharmacists’ roles and responsibilities in this model. | ☐ | ☐ | ☐ | ☐ | ☐ |
| 25. Legal and professional liability in remote dispensing is unclear. | ☐ | ☐ | ☐ | ☐ | ☐ |
| 26. There is an absence of standardized protocols for prescription verification, documentation, and pharmacist counselling. | ☐ | ☐ | ☐ | ☐ | ☐ |
| 27. Current technological infrastructure is insufficient to support safe telemedicine-enabled dispensing. | ☐ | ☐ | ☐ | ☐ | ☐ |
| 28. There is a lack of standardized medication delivery protocols ensuring safe handling, storage, and traceability. | ☐ | ☐ | ☐ | ☐ | ☐ |
| 29. This model may disrupt established pharmacy workflow. | ☐ | ☐ | ☐ | ☐ | ☐ |
| 30. This model may increase administrative burden on pharmacists. | ☐ | ☐ | ☐ | ☐ | ☐ |
| 31. This model may negatively affect the sustainability of community pharmacies. | ☐ | ☐ | ☐ | ☐ | ☐ |
| 32. Overall, major implementation barriers currently limit safe adoption of this model. | ☐ | ☐ | ☐ | ☐ | ☐ |

**Scale:** 1 = Strongly disagree, 2 = Disagree, 3 = Neutral/Unsure, 4 = Agree, 5 = Strongly agree.

**Section E. Facilitators and safeguards for safe implementation**

| **Item** | **1** | **2** | **3** | **4** | **5** |
| --- | --- | --- | --- | --- | --- |
| 33. Mandatory pharmacist review and verification of all prescriptions before dispensing would improve my confidence in this model. | ☐ | ☐ | ☐ | ☐ | ☐ |
| 34. A clear regulatory framework with legal protections for pharmacists would improve acceptance. | ☐ | ☐ | ☐ | ☐ | ☐ |
| 35. Clearly defined legal liability structures would improve my willingness to participate. | ☐ | ☐ | ☐ | ☐ | ☐ |
| 36. Standardized operating procedures for verification, documentation, and counselling would improve implementation feasibility. | ☐ | ☐ | ☐ | ☐ | ☐ |
| 37. Mandatory direct pharmacist–patient counselling (e.g., phone or video) before dispensing would improve safety. | ☐ | ☐ | ☐ | ☐ | ☐ |
| 38. Temperature-controlled delivery systems and standardized medication handling protocols would improve safety. | ☐ | ☐ | ☐ | ☐ | ☐ |
| 39. A secure integrated electronic prescription system with audit trails would improve acceptance. | ☐ | ☐ | ☐ | ☐ | ☐ |

**Scale:** 1 = Strongly disagree, 2 = Disagree, 3 = Neutral/Unsure, 4 = Agree, 5 = Strongly agree.

**Section F. Optional additional comments**

**40. What is the main reason for your current level of acceptance or resistance?**

________________________________________________________________________________

________________________________________________________________________________

**41. What do you think is the most important safeguard required before implementation?**

________________________________________________________________________________

________________________________________________________________________________

**42. Any additional comments or recommendations:**

________________________________________________________________________________

________________________________________________________________________________
